# Motor Imagery for Post-Stroke Upper Limb Recovery: A Meta-Analysis of RCTs on Fugl-Meyer Upper Extremity Scores

**DOI:** 10.3390/jcm14217891

**Published:** 2025-11-06

**Authors:** Luis Polo-Ferrero, Javier Torres-Alonso, Juan Luis Sánchez-González, Sara Hernández-Rubia, Rubén Pérez-Elvira, Javier Oltra-Cucarella

**Affiliations:** 1 Department of Nursing and Physiotherapy, Universidad de Salamanca, 37007 Salamanca, Spain; javiertorres@usal.es (J.T.-A.); sarahernandezrubia@usal.es (S.H.-R.); 2 Instituto de Investigación Biomédica de Salamanca (IBSAL), 37007 Salamanca, Spain; juanluissanchez@usal.es; 3 Department of Medicine, Universidad de Salamanca, 37007 Salamanca, Spain; 4 Department of Psychobiology, Pontifical University of Salamanca, 37002 Salamanca, Spain; rperezel@upsa.es; 5 Laboratory of Neuropsychophysiology, NEPSA Rehabilitación Neurológica, 37003 Salamanca, Spain; 6 Department of Health Psychology, Universidad Miguel Hernández de Elche, 03202 Alicante, Spain

**Keywords:** motor imagery, stroke, upper-limb recovery, Fugl-Meyer, neurorehabilitation, meta-analysis

## Abstract

**Objectives**: Motor imagery (MI) may enhance post-stroke recovery, but evidence of its benefit over conventional rehabilitation therapy (CRT) is inconsistent. This study evaluated the effect of MI combined with CRT on upper-limb recovery, accounting for methodological quality and publication bias. **Methods**: A systematic review and meta-analysis was conducted following PRISMA guidelines. Searches were performed in multiple databases up to July 2025. Methodological quality and risk of bias were assessed using the PEDro scale and Cochrane RoB 2 tool, respectively. Analyses included the calculation of effect sizes (ES), heterogeneity, sensitivity, publication bias, and GRADE-based certainty assessment. **Results**: From 4074 records, 10 randomized controlled trials (*n* = 255) were included. The initial pooled analysis showed a small-to-moderate effect of MI + CRT versus CRT alone (ES = 0.45; 95% CI: 0.16–0.74). However, the overall ES calculated with a robust variance estimator was −0.06 (95% CI: −0.21, 0.08). Most trials had methodological limitations (mean PEDro = 6.0; high risk of bias in 7/10 studies). The GRADE evaluation indicated a very low certainty of evidence. **Conclusions**: The initially observed positive effect of MI combined with CRT is not robust. When accounting for statistical dependencies and potential biases, the effect vanishes and is no different from zero. Current evidence does not support the use of MI as a standalone adjunct to CRT. Larger, high-quality RCTs with standardized protocols are required to establish any potential clinical relevance.

## 1. Introduction

Stroke remains a leading cause of acquired adult disability worldwide, with motor impairment of the upper limb representing one of its most frequent and devastating consequences. The functional use of the arms and hands is fundamental to the performance of essential activities of daily living, such as eating, dressing, and personal hygiene. Persistent upper limb deficits—including spasticity, loss of fractionated movement, abnormal synergies, and reduced strength and dexterity—directly compromise a survivor’s autonomy, social participation, and overall quality of life [1,2,3]. The profound impact of this motor disability underscores the critical need for effective rehabilitation strategies that can translate into meaningful functional gains.

The accurate and sensitive measurement of motor recovery is therefore paramount for both clinical practice and research. A variety of tools exist to assess upper limb function post-stroke, ranging from capacity-based measures that evaluate performance in a clinical setting to performance-based measures that capture real-world arm use. These include the Action Research Arm Test (ARAT), the Wolf Motor Function Test (WMFT), and the Motor Activity Log (MAL), among others [4,5,6]. However, the Fugl-Meyer Assessment for the upper extremity (FM-UE) has emerged as the gold standard for quantifying specific sensorimotor recovery [7,8,9]. Its comprehensive nature, which systematically assesses reflex activity, volitional movement within and outside synergies, coordination, and speed, provides a granular picture of motor impairment that aligns closely with underlying neurophysiological processes. Furthermore, its excellent reliability, high responsiveness to change, and strong convergent validity with other motor and functional scales make it a robust and widely accepted primary endpoint for clinical trials focused on neurological recovery mechanisms [7,8,9].

Despite advances in rehabilitation, functional outcomes remain suboptimal, with fewer than 20% of patients achieving full upper limb recovery through conventional physical and occupational therapy alone [10,11,12]. This persistent therapeutic gap has driven the exploration of complementary, evidence-based interventions. Among these, motor imagery (MI), or mental practice (MP), has gained traction as a promising cognitive-kinesthetic approach. MI is defined as the internal simulation and rehearsal of motor actions without their overt physical execution [2]. The rationale for its application in neurorehabilitation is grounded in neurophysiological evidence demonstrating that MI activates a network of brain regions—including the premotor cortex, supplementary motor area, inferior parietal lobule, and cerebellum—that substantially overlaps with the neural substrates engaged during actual movement execution [13]. This shared neural circuitry is believed to facilitate use-dependent neuroplasticity, a cornerstone of motor recovery. Given that high-intensity, repetitive task practice is a key driver of this plasticity [14], MI presents a feasible, low-cost, and accessible method to augment physical training, potentially overcoming logistical and financial barriers while promoting functional reorganization in the brain.

Several randomized clinical trials (RCTs) have investigated the efficacy of MI, reporting encouraging yet inconsistent results. Previous meta-analyses have provided valuable insights but were limited by factors such as heterogeneous control groups (CG), small sample sizes, the inclusion of non-randomized studies, and literature searches that are now outdated, with the most recent comprehensive search concluding in 2021 [15,16,17]. Furthermore, the field lacks standardized MI protocols, with critical parameters like optimal dosing, session duration, and timing of delivery remaining poorly defined [18]. Although one study demonstrated a dose–response relationship between MI “training load” and FM-UE improvements [19], and others suggest potential benefits across both subacute and chronic phases [17], a direct, rigorous comparison of effect sizes is lacking. By explicitly addressing these prior limitations, the present study clearly positions itself within the existing body of evidence and defines its added methodological value.

This meta-analysis therefore aims not only to update the evidence base but also to address prior limitations through stricter eligibility criteria and advanced statistical methods. By exclusively comparing MI combined with conventional rehabilitation therapy (CRT) against CRT alone, this review seeks to quantify the specific impact of MI on the FM-UE. Robustness will be ensured through comprehensive sensitivity analyses, rigorous assessment of publication bias, and moderator analyses to explore the influence of study quality, patient characteristics, and intervention parameters. This methodological framework aims to deliver a more reliable and clinically relevant synthesis, strengthening the evidence base and clarifying the role of MI in post-stroke rehabilitation.

## 2. Materials and Methods

### 2.1. Data Sources and Search Strategy

The review process adhered to the PRISMA statement for systematic reviews [20], and the corresponding PRISMA 2020 checklist is provided in Supplementary Material S1. The protocol was prospectively registered in PROSPERO (registration ID: CRD420251120044). To identify eligible studies, we carried out a structured search across PubMed, Cochrane Library, CINAHL, Scopus, Web of Science, and ScienceDirect, covering all records available up to July 2025. Search terms and Boolean combinations were adapted to the characteristics of each database, and the full strategies are presented in Supplementary Material S2.

### 2.2. Eligibility Criteria

Eligibility criteria were defined according to the PICOS framework (Population, Intervention, Comparison, Outcomes, and Study design) [21], with no restrictions on publication year.

Inclusion criteria were as follows:

(1) adult participants with a clinical diagnosis of stroke presenting upper-limb motor impairment, regardless of stroke type, severity, or recovery phase;
(2) interventions combining MI or MP with CRT;
(3) control groups receiving the same CRT protocol as the intervention group, without MI;
(4) upper-limb motor outcomes assessed through the FM-UE scale; and
(5) RCTs, including pilot or crossover RCTs.

Exclusion criteria included:

(1) studies combining MI with other non-conventional interventions (e.g., virtual reality, mirror therapy, or brain–computer interfaces);
(2) studies not reporting sufficient data for effect size calculation; and
(3) non-randomized, quasi-experimental, or single-case designs.

### 2.3. Outcome Measures

The FM-UE was selected as the primary outcome given its strong psychometric properties and clinical relevance. FM-UE is the most frequently applied subscale of the FM for post-stroke evaluation, with a maximum score of 66 points. It assesses upper-limb sensorimotor function, covering movement within and outside synergies, reflex activity, isolated joint control, coordination, wrist and hand function, and movement speed. Items are scored on a 3-point ordinal scale (0 = cannot be performed, 1 = partially performed, 2 = fully performed), with higher scores reflecting greater motor recovery and functional capacity. Interpretation of total scores classifies impairment severity as severe (≤28), moderate (29–42), mild (43–52), or near-normal (≥53). The FM-UE demonstrates excellent reliability (ICC = 0.89–0.99 across studies), strong criterion validity through high correlations with other motor assessments, including the ARAT (r = 0.93) and the Box and Block Test (BBT, r = 0.86), and high responsiveness to change, with standardized response mean values typically exceeding 0.8 in stroke rehabilitation studies, confirming its sensitivity to clinically meaningful improvement [9,22]. Although methodological heterogeneity exists across studies, evidence consistently supports the FM-UE as a gold-standard instrument for assessing post-stroke upper-extremity sensorimotor recovery, with extensive validation and widespread clinical and research use [6].

### 2.4. Study Selection

Two independent reviewers (L.P.-F. and J.T.-A. conducted the literature screening using Rayyan software (https://www.rayyan.ai/, accessed on 15 July 2025), following the same predefined methodology. Titles and abstracts retrieved from the search were first assessed to exclude irrelevant records. Full-text articles of potentially eligible studies were then reviewed in detail to determine final inclusion. Any discrepancies between reviewers were resolved by consensus with a third reviewer (JLSG). In addition, the reference lists of the included studies were manually examined to identify further relevant publications, and corresponding authors were contacted to obtain missing or clarifying information when required.

### 2.5. Data Extraction

Data extraction was conducted independently following a pre-defined protocol, and all extracted information was cross-checked to ensure accuracy and completeness. For each study, the following information was collected: study design, sample size, participant characteristics (age, sex, stroke phase, stroke type [ischemic or hemorrhagic], lesion laterality, and baseline severity assessed with FM-UE), group allocation, and intervention details (type of motor imagery, content of conventional therapy, frequency [sessions per week], session duration in minutes, and total intervention length in weeks). Reported outcomes and main findings were also registered. Pre- and post-intervention means and standard deviations were extracted for both intervention and control groups. In addition, the pre–post correlation within each group was considered for the calculation of effect sizes. For studies reporting individual-level data, correlations were directly computed [23], whereas for those providing only aggregated data, a conservative correlation coefficient of 0.89 was applied based on the FM-UE pre-post correlation reported by Philips et al. [24].

### 2.6. Risk of Bias and the Assessment of Methodological Quality of the Studies

The internal validity of the included trials was evaluated with the revised Cochrane Risk of Bias tool for randomized trials (RoB 2), including the specific adaptation for crossover trials when applicable [25]. These domains are grounded in both empirical evidence and theoretical rationale. For parallel-group randomized controlled trials, this instrument evaluates five potential sources of bias: the randomization process, deviations from intended interventions, missing outcome data, outcome measurement, and selective reporting of results. In the case of crossover trials, additional considerations were included, such as the potential for carryover effects and the appropriateness of the analysis in the context of a two-period design. Each study was rated as presenting a low risk of bias, some concerns, or high risk of bias, depending on the level of concern identified across these domains. The RoB 2 assessment was independently conducted by two reviewers. Inter-rater agreement between the two reviewers was assessed using Cohen’s kappa statistic for each domain and for the overall risk of bias judgment [25]. The strength of agreement was interpreted as follows: values ≤ 0 indicating no agreement, 0.01–0.20 as slight, 0.21–0.40 as fair, 0.41–0.60 as moderate, 0.61–0.80 as substantial, and 0.81–1.00 as almost perfect agreement [26]. Any discrepancies between reviewers were resolved through discussion with a third reviewer to reach a final consensus rating for each domain and overall risk of bias.

Additionally, the methodological quality of the studies was assessed using the PEDro scale [27] which evaluates both internal and external validity based on 11 criteria: (1) clearly defined eligibility criteria; (2) random allocation of participants; (3) concealed allocation; (4) baseline comparability of groups; (5) participant blinding; (6) therapist blinding; (7) outcome assessor blinding; (8) attrition below fifteen percent; (9) intention-to-treat analysis; (10) statistical comparisons between groups; and (11) reporting of point estimates together with variability measures. Criterion (1) is required for completeness of the checklist, but it is not included in the calculation of the final PEDro score. Each criterion was scored as either “yes” (1 point), “no” (0 points), or “unclear” (0 points). The PEDro score was used to complement the RoB 2 assessment, providing a broader evaluation of the methodological quality of the included trials. The overall PEDro score for each study served as an indicator of its methodological quality: 9–10 = excellent; 6–8 = good; 4–5 = fair; and 0–3 = poor [28].

### 2.7. Overall Quality of Evidence

The certainty of evidence regarding MI in post-stroke patients for FM-UE was assessed using the GRADE system (Grading of Recommendations Assessment, Development and Evaluation) [29]. It classifies evidence into four levels (high, moderate, low, or very low) based on risk of bias, inconsistency, indirectness, imprecision, and publication bias. High-quality evidence indicates solid confidence in the effect estimate, while very low-quality evidence reflects minimal confidence in the observed results [30].

### 2.8. Studies Data Synthesis and Analysis

Meta-analysis was performed with R software (version 2024.12.0.467) [31]. Since the effect size (ES) of interest was the pre/post difference in the primary outcome between groups (intervention vs. control) within studies, a multivariate model was used with the package metafor [32]. The Standardized Mean Change (SMC) using raw scores was calculated with the function escalc(), which calculates the ES for each group within each study as $\frac{{µ}_{pre}-\;{µ}_{post}}{{sd}_{pre}}$, where ${µ}_{pre}$ is the mean at pretest (baseline), ${µ}_{post}$ is the mean at posttest (after the intervention), and ${sd}_{pre}$ is the standard deviation at pretest. Since the calculation of the variance of the SMC requires the correlation between pre and post scores, we calculate the point biserial correlation in primary studies where available, and missing values were imputed with an r = 0.894 as reported by Philips, Daly and Principe [24]. Individual ESs within studies were inverted for positive values to indicate higher scores (i.e., improvement of the underlying construct) at posttest. A model was then fitted with the rma.mv() function allowing for a random intercept for each individual study included in the meta-analysis, with the restricted maximum likelihood (REML) method for estimating heterogeneity. After calculating the ESs per group within studies, a single ES per study was calculated with the function aggregate(), with a variance-covariance structure calculated from the function detailed above. ESs were weighted by the inverse of their variance, which included the sampling variance, the variance between studies (t^2^) and a certain amount of covariance between the effects [33]. Following conventional guidelines, the overall ES are interpreted as low, medium and large for values 0.20–0.49, 0.50–0.79, and ≥0.80, respectively [34]. All extracted data used for the analyses are provided in Supplementary Material S3, along with the complete R code to ensure full reproducibility.

#### 2.8.1. Heterogeneity in ES Estimates

The orchaRd package [35] was also used to calculate the I^2^ statistic, which measures the proportion of total variation in study estimates that is due to heterogeneity [36], with values of 25%, 50% and 75% representing low, medium and high heterogeneity, respectively [37].

#### 2.8.2. Sensitivity Analysis

A leave-one-out sensitivity analysis was carried out with the function leave_one_out() from the orchaRd package [35] in order to calculate variations in the overall ES when each individual study was removed one at a time, and the results were plotted with the function orchard_leave1out().

#### 2.8.3. Publication Bias Analysis

To control for how selective reporting could affect the overall ES estimate, we calculated the variation in the overall ES estimate due to publication bias with the function pub_bias_plot () from the orchaRd package. To do so, we fitted different models in several steps, as per the method developed by Yang et al. [38]. In step 1, we fitted an intercept only fixed effect model with two (one for control and one for experimental groups) effect sizes and variances within each primary study. In step 2, we fitted a model that controlled for dependency of effect sizes within clusters (i.e., primary studies) using a cluster robust variance estimation of the model coefficients. In step 3, we used a multilevel Egger’s test by modeling the sampling variance of each individual ES as a predictor [39], with random intercept for each study, and the difference in the overall ES estimate was plotted. 

#### 2.8.4. Moderator Analyses

We ran a series of meta-regressions to analyze the influence of age, sex, and the quality of studies on the overall ES. Scores on the PEDro scale, the RoB scale, the phase (subacute phase as the reference), the frequency of the intervention, the number of sessions, and the duration of the sessions were modeled as predictors of the overall ES in univariate models due to the small sample size. For the analyses of the RoB scale the category with small risk of bias was taken as the reference, since studies with higher risk of bias tend to report higher ESs [40,41].

All analyses for estimating ESs were computed with a t-distribution for coefficients and confidence intervals. The script with the code used to conduct all the analysis is available at the author’s website [42].

## 3. Results

### 3.1. Search Results and Study Selection

**Figure 1:**
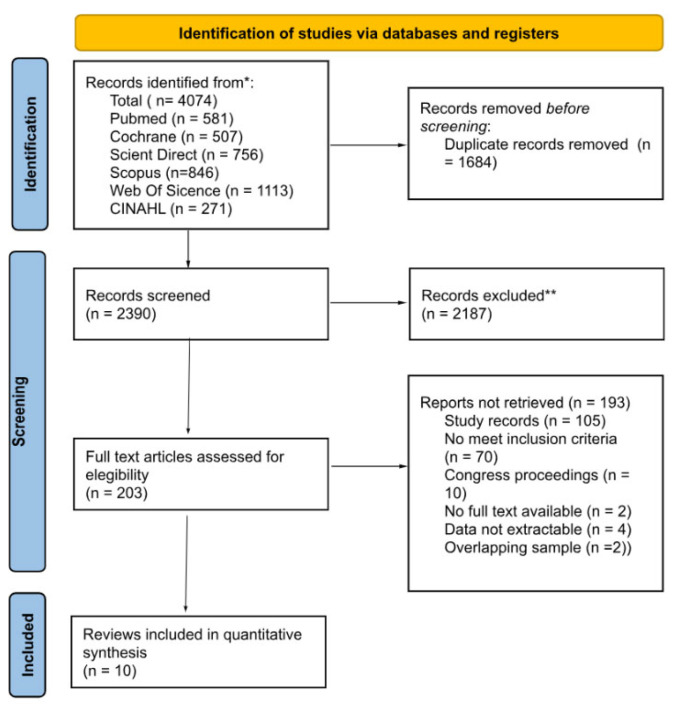
Flow diagram of the study selection process according to the PRISMA guidelines. * Records identified from: PubMed, Cochrane Library, Sciencedirect, Scopus, Web of Science, and CINAHL databases. ** Records excluded: Articles removed after title and abstract screening because they did not meet the predefined inclusion criteria (e.g., population, intervention, outcomes, or study design).

The search strategy identified a total of 4074 records from different databases (PubMed *n* = 581, Cochrane *n* = 507, Science Direct *n* = 756, Scopus *n* = 846, Web of Science *n* = 1113, and CINAHL *n* = 271). After removing 1684 duplicates, 2390 records were screened by title and abstract, of which 2187 were excluded for not meeting the inclusion criteria. A total of 203 full-text articles were assessed for eligibility. Of these, 193 reports were excluded: 105 were study records, 70 did not meet the inclusion criteria, 10 were congress proceedings, 2 were not available in full text, 4 did not provide extractable data, and 2 presented overlapping samples. Ultimately, 10 RCTs were included in the review and meta-analysis. The entire selection process is summarized in the PRISMA flow diagram (Figure 1).

### 3.2. Study Characteristics

A total of 255 participants were included across the 10 studies, with 131 assigned to the intervention groups (mean age: 57.3 ± 4.7 years; 26.5% women) and 124 to the control groups (mean age: 58.8 ± 3.8 years; 24.3% women). The majority of trials were RCTs (*n* = 8), complemented by one pilot RCT [43] and one crossover design [44]. The mean sample size per study was 13.1 ± 4.9 participants in the intervention group (IG) and 12.4 ± 4.6 in the CG.

Regarding stroke chronicity, three studies enrolled patients in the subacute phase [43,44,45], five studies focused on chronic stroke survivors [46,47,48,49,50] and two included mixed samples of subacute and chronic phases [51,52]. Lesion laterality was reported in all studies except one study Nayeem et al. 2012 [50], with both hemispheres represented across samples. Distribution was balanced between groups (IG: 6.3 ± 3.0 left hemisphere, 6.2 ± 2.7 right hemisphere; CG: 6.4 ± 2.2 left hemisphere, 2.9 right hemisphere).

Stroke type varied among studies: six trials included both ischemic and hemorrhagic stroke [43,44,45,48,49,52], three did not specify stroke type [46,47,50], and one excluded hemorrhagic cases, including only ischemic patients [51].

The majority of intervention groups were classified as having severe baseline deficits (mean FM-UE: 14.9–22.44; *n* = 5 studies) [43,45,48,49,52], followed by moderate (29.1–42; *n* = 3 studies) [46,47,51]. A single study enrolled participants with near-normal motor function (mean FM-UE: 58.0) [50], while another included a cohort with mild impairment (mean FM-UE: 46.4) [44]. This distribution was well-balanced between intervention and control arms within each trial.

Intervention parameters were heterogeneous. The mean duration of MI interventions was 3.9 ± 1.5 weeks, with an average frequency of 4.2 ± 1.4 sessions per week, totaling 15.6 ± 5.6 sessions. CRT sessions provided alongside MI varied considerably, with an average of 85.5 ± 71.4 min per session, ranging from 15 min [50] to 180 min [48,49,52]. MI sessions lasted on average 22.5 ± 8.6 min, leading to a cumulative exposure of 375 ± 216.8 min per participant. Additional study characteristics and intervention details are presented in Table 1.

### 3.3. Meta-Analysis of the Effects of MI on Motor Recovery

**Figure 2:**
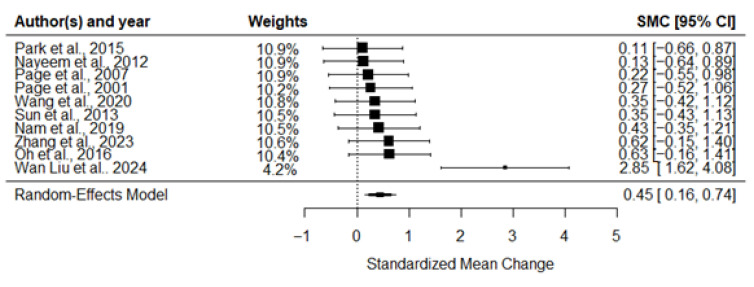
Forest plot for the overall effect size. The analysis includes the following studies: Nam et al., 2019 [43]; Nayeem et al., 2012 [50]; Oh et al., 2016 [44]; Page et al., 2001 [51]; Page et al., 2007 [46]; Park et al., 2015 [47]; Sun et al., 2013 [48]; Wan Liu et al., 2024 [45]; Wang et al., 2020 [49]; Zhang et al., 2023 [52].

The meta-analysis showed a statistically significant small to moderate overall ES = 0.45 (*n* = 10, k = 20, df = 19, se = 0.13, 95%CI: 0.18–0.72, *p* = 0.003), as shown in Figure 2 favoring the intervention groups. The I^2^ statistic showed high heterogeneity in effect sizes between studies (I^2^ = 88.2%). The overall ES was not affected by sex (b = 1.40, 95%CI: −1.69, 4.49, *p* = 0.319) or age (b = −0.01, 95%CI: −0.18, 0.16, *p* = 0.881).

### 3.4. Methodological Quality

Across the 10 included studies, PEDro scores ranged from 5 to 7, with a mean score of 6.0, reflecting overall moderate methodological quality. Four studies scored 5 points [45,47,48,49], four studies scored 6 points [43,44,50,51], and two studies reached 7 points [46,52]. Most studies adequately reported randomization, baseline comparability, between-group comparisons, and variability measures. The main limitations were lack of concealed allocation and blinding of participants and therapists, while assessor blinding was reported in six studies. Detailed PEDro scores for each study are presented in Table 2.

### 3.5. Risk of Bias

**Figure 3:**
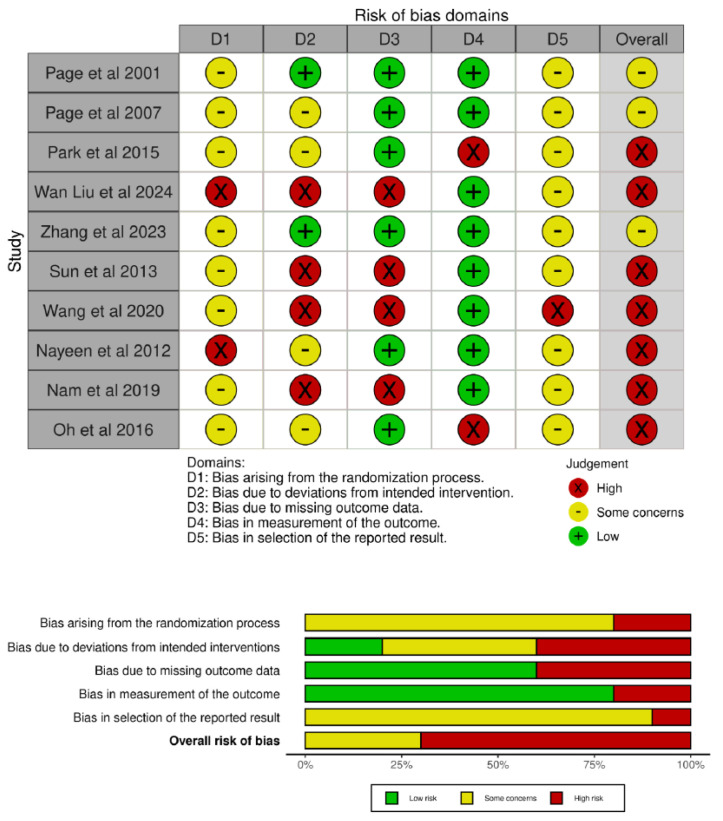
Risk of bias assessment for the included studies [43,44,45,46,47,48,49,50,51,52].

The risk of bias assessment was performed using the Cochrane RoB 2 tool for randomized parallel trials for nine studies, and the RoB 2 for crossover trials for one study [44]. Inter-rater agreement between reviewers, assessed using Cohen’s kappa prior to consensus, was 0.78, indicating a moderate-high level of agreement. The overall judgements are summarized in Figure 3.

Three RCTs [46,51,52] raised “Some Concerns” due to inadequate reporting of randomization procedures and lack of blinding of participants and personnel—a limitation inherent to the intervention that may have introduced performance bias.

Six parallel RCTs [43,45,47,48,49,50] were judged at “High Risk” of bias, primarily due to lack of blinding leading to deviations from intended interventions, and high attrition rates or inadequate application of intention-to-treat analysis introducing bias from missing outcome data.

The crossover trial [44] was also assessed as “High Risk”. The primary concern was the potential for carryover effects between treatment periods, which were not adequately accounted for in the study design or statistical analysis. In addition, both domain 1 (randomization process) and domain 1b (period and carryover effects) were judged as “Some Concerns”; however, for clarity, these were unified in Figure 3.

### 3.6. Overall Quality of Evidence

The certainty of the evidence was assessed using the GRADE framework and was downgraded due to a very serious risk of bias, serious imprecision, and very serious publication bias, resulting in an overall very low certainty (Table 3).

### 3.7. Sensitivity Analyses

**Figure 4:**
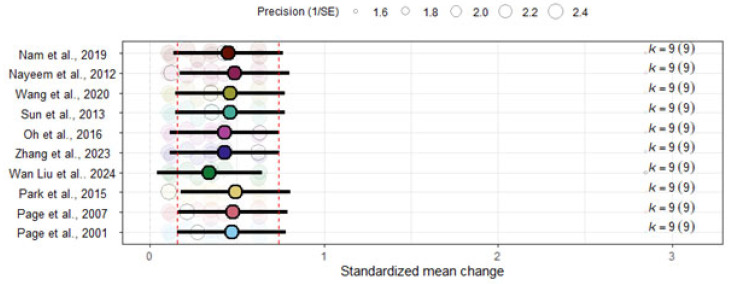
Leave-one-out sensitivity analysis of overall effect size. The analysis includes the following studies: Nam et al., 2019 [43]; Nayeem et al., 2012 [50]; Oh et al., 2016 [44]; Page et al., 2001 [51]; Page et al., 2007 [46]; Park et al., 2015 [47]; Sun et al., 2013 [48]; Wan Liu et al., 2024 [45]; Wang et al., 2020 [49]; Zhang et al., 2023 [52].

The leave-one-out analysis showed that the overall ES remained unchanged, with every overall ESs after removal of one study falling within the 95% confidence interval of the overall ES when all studies were analyzed (Figure 4).

### 3.8. Publication Bias Analyses

**Figure 5:**
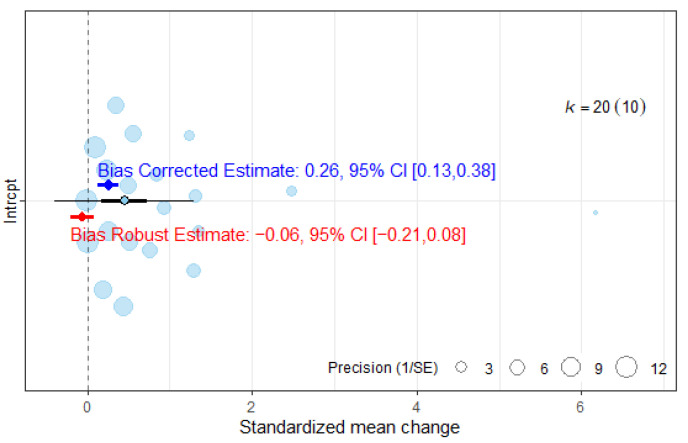
Publication bias analysis of overall effect size.

The publication bias analyses (Figure 5) showed that the overall ES would become smaller than the ES found in the absence of selective reporting (ES = 0.26, 95%CI: 0.13, −0.38). The overall ES calculated with robust variance estimator was −0.06 and not statistically different from zero (95%CI: −0.21, 0.08).

### 3.9. Moderator Analyses

The meta-regression using the PEDro scale as a moderator showed that the overall ES was not affected by the methodological quality of the primary studies (b = −0.15, 95%CI: −0.69, 0.39, *p* = 0.544), nor was it statistically significantly higher in studies with high risk of bias according to the RoB scale (b = 0.17, 95%CI: −0.68, 1.02, *p* = 0.656).

Additionally, the overall ES was not affected by the phase of the disease (b = −0.30, 95%CI: −0.83, 0.22, *p* = 0.223), the degree of severity of the motor deficit at baseline (all *p*-values > 0.382), the frequency of the intervention (b = 0.11, 95%CI: −0.24, 0.47, *p* = 0.472), the number of sessions delivered (b = 0.03, 95%CI: −0.03, 0.09, *p* = 0.267) or the duration of the MI intervention (all *p*-values > 0.370).

## 4. Discussion

This meta-analysis provides a quantitative synthesis of the additive effect of MI to CRT on upper limb motor recovery, measured by the FM-UE, across 10 RCTs. The overall small-to-moderate effect size with negligible heterogeneity suggests a potential benefit of adjunctive MI. However, considering methodological weaknesses and publication bias, this apparent effect should be interpreted cautiously.

### 4.1. Interpretation of Findings and Clinical Relevance

The apparent positive effect of MI must be interpreted in light of methodological weaknesses. Risk of bias assessment (RoB 2) showed that 7 of 10 trials were at high risk, mainly due to the impossibility of blinding participants and therapists and incomplete outcome data, both known to inflate effects in rehabilitation research [53]. Although meta-regressions using PEDro and RoB scores did not reveal significant moderating effects, this likely reflects limited statistical power (k = 10) rather than absence of bias. The consistent direction of potential bias suggests an overestimation of the treatment effect.

The most critical finding of this review comes from the publication bias analysis. When selective reporting of positive results was modeled, the adjusted overall effect decreased slightly, but the robust variance estimator showed that the overall ES became statistically non-significant. Consequently, the evidence is insufficient to conclude that MI provides a clinically meaningful benefit over CRT alone. The observed positive effect is likely an overestimate, and adjusted models suggest that the true effect may be small or even negligible. Considering the very low certainty of evidence according to GRADE, these findings should be interpreted with caution [54].

As in other scientific fields where potential increases in scores on cognitive tests do not seem to correlate with functional improvements [55], the clinical relevance of the unadjusted effect size is questionable. While this value meets the threshold for a small-to-moderate effect, when translated into FM-UE points it falls below the minimal clinically important difference (MCID), estimated at 12.4 points for the upper extremity subscale [56]. This reference was chosen because the majority of participants in the included studies presented moderate or severe hemiparesis, and only one study included patients with mild impairment. However, MCID estimates may vary depending on stroke severity and methodology, ranging from 5 to 11 points according to See et al. (2013) [57]. Only five studies reported mean improvements exceeding this MCID threshold [45,48,49,51,52], with the ES indicating a 4.94 points main gain on average from baseline scores across studies. Notably, three of these five trials were at high risk of bias, and four included cohorts with severe baseline impairment (FM-UE ≤ 19). This pattern is consistent with two phenomena: the ceiling effect of the FM-UE scale, limiting measurable improvement in patients with mild-to-moderate impairment, and the principle that patients with greater initial deficits have larger potential for absolute recovery, disproportionately influencing effect estimates [57,58], However, subgroup analysis based on baseline severity did not show a statistically significant superior effect of MI in the severe cohort. Thus, the observed positive aggregate effect seems to be driven by biased and non-generalizable studies.

Beyond these quantitative findings, an important aspect concerns the neurobiological and neurophysiological correlates of MI efficacy. Recent reviews highlight the interplay between the cerebellum and the mirror neuron system, particularly through inhibitory mechanisms relevant to motor relearning. Given the cerebellum’s central role in post-stroke recovery and cortical reorganization, MI may engage these interconnected circuits [59]. However, whether such neural activation translates into measurable functional improvement remains unclear, underscoring the need to integrate behavioral and neuroimaging markers in future studies to bridge this mechanistic–clinical gap.

When compared with other pathological contexts, MI results in stroke appear less consistent. In chronic pain and multiple sclerosis, MI—or its variants such as graded motor imagery—has shown moderate functional improvements, though supported by methodologically weak evidence. In Parkinson’s disease, action observation therapy (AO) and its combination with MI seem more effective, likely due to the visual feedback and external cueing that facilitate motor activation [60,61,62]. In contrast, in stroke, MI alone does not yield clinically relevant effects once bias is accounted for, suggesting that its efficacy depends on the pathological context and may require integrating strategies that compensate for limitations in internal movement generation.

### 4.2. Methodological Considerations and Limitations

A major limitation lies in the heterogeneity of intervention protocols and participant characteristics. The content of CRT was not standardized across studies, reflecting differences in clinical practice and healthcare resources. Although both IG and CG received the same baseline CRT, variations in what was considered “conventional rehabilitation” may have influenced treatment response. Future trials should provide detailed descriptions of CRT to improve comparability. However, the aim of this meta-analysis was not to compare rehabilitation approaches, but to assess whether adding MI to CRT—regardless of its composition—enhances motor recovery.

Key MI parameters (duration, frequency, total dose, and mode of guidance) varied considerably, as did stroke chronicity and baseline impairment. Essential details—such as whether imagined movements were transitive or intransitive, the use of rest periods, or strategies to sustain attention—were rarely reported. This lack of methodological detail limits interpretation and prevents identifying optimal MI features, dosing, or the most effective recovery phase. One study suggested a dose–response trend between longer MI training and greater FM-UE gains [19], although no trial achieved improvements surpassing the MCID threshold. The absence of phase-specific effects may reflect persistent neuroplasticity long after stroke [63]. Moreover, incomplete reporting of stroke recurrence contributed to clinical heterogeneity, as recurrent strokes typically entail greater impairment and slower recovery.

Another critical gap is the lack of screening for MI ability before inclusion. Only two studies excluded participants with poor imagery capacity [45,51]. ince imagery ability is often impaired after stroke and predicts recovery potential [64,65], failing to assess it likely diluted true effects by including non-responders. Future trials should systematically evaluate MI ability using validated tools such as mental chronometry or standardized questionnaires to ensure appropriate selection.

Overall, heterogeneity in interventions, small sample sizes, short durations, and absence of long-term follow-up highlight the need for more rigorous research. Larger, adequately powered RCTs are required to provide reliable estimates. Moreover, reaching consensus on MI dosing parameters—frequency, intensity, duration, and cumulative exposure—is essential to improve reproducibility and clinical translation. Greater methodological rigor and standardization will enhance precision and strengthen the applicability of MI as a potential adjunctive therapy.

### 4.3. Future Research Directions

Future research should prioritize methodological rigor and standardization to strengthen the evidence base on motor imagery in stroke rehabilitation. Establishing standardized MI protocols that clearly define session frequency, intensity, duration, and total dose is essential to improve comparability and reproducibility. Trials should also include baseline assessments of MI ability using validated tools to ensure inclusion of participants capable of accurate motor representation, thereby reducing variability and improving validity. Adequately powered RCTs with long-term follow-up are needed to confirm the persistence of benefits and identify subgroups most likely to respond. Reporting null or negative results remains crucial to mitigate publication bias and enhance the reliability of pooled estimates.

Advances in neurorehabilitation technologies offer promising avenues to enhance MI efficacy. Combining MI with immersive virtual or augmented reality could strengthen motor network activation and bridge the gap between mental representation and physical execution [66,67]. Similarly, integrating MI into closed-loop brain–computer interfaces coupled with functional electrical stimulation (BCI-FES), often supported by robotics, may promote cortical reorganization and neuroplasticity [68,69]. The combination of MI with non-invasive brain stimulation (NIBS) techniques—such as transcranial direct current stimulation (tDCS) or repetitive transcranial magnetic stimulation (rTMS)—also shows potential to enhance cortical excitability and motor learning, improving upper-limb recovery [70].

Future trials should also integrate multimodal biomarkers, such as EEG combined with kinematic or hemodynamic analyses using functional near-infrared spectroscopy (fNIRS), to objectively monitor MI-induced changes and reduce reliance on clinical scales. Recent multimodal approaches using EEG–fNIRS [71] and EEG–kinematic integration [72] have demonstrated complementary insights into post-stroke functional status and potential response biomarkers [59]. Moreover, event-related desynchronization (ERD) during AO has emerged as an early predictor of recovery in subcortical stroke. Given the shared neural substrates between AO and MI, ERD-AO and cortico-cerebellar connectivity metrics could help stratify participants and identify MI responders. Embedding these biomarkers as a priori moderators and secondary endpoints may clarify dose–response relationships, underlying mechanisms, and patient profiles most likely to benefit from MI.

### 4.4. Clinical Implications and Final Remarks

Fewer than 20% of stroke survivors achieve full upper limb recovery, and only 30–40% regain useful hand and arm function within six months post-stroke [10,73]. Against this backdrop, current evidence indicates that while MI combined with CRT may offer potential benefits, its clinical efficacy remains uncertain, and routine implementation in standard rehabilitation cannot yet be recommended. Still, available data outline features of a potentially optimal MI protocol for future trials: structured, therapist-guided sessions integrated within CRT rather than delivered in isolation. Sessions lasting 20–30 min, 3–5 times per week over 4–6 weeks, appear feasible and consistent with the most favorable trends. Using first-person, kinesthetic imagery focused on goal-directed, task-specific actions may further enhance cortical activation and functional relevance.

Regarding patient selection, individuals with preserved cognitive and attentional abilities, mild-to-moderate motor impairment, and demonstrable imagery capacity are most likely to benefit, whereas those with severe cognitive, perceptual, or language deficits may not. Systematic pre-assessment of MI ability using validated tools is therefore recommended to optimize patient selection and intervention fidelity.

The persistent limitations of standard rehabilitation underscore the need for rigorously designed RCTs that standardize MI parameters and identify responders based on clinical and neurocognitive profiles. Determining whether optimized MI protocols can meaningfully enhance upper-limb motor recovery and establishing evidence-based criteria for patient selection will be essential to bridge the gap between theoretical promise and clinical applicability. These efforts are critical to advance toward more effective, targeted, and clinically relevant rehabilitation strategies.

## 5. Conclusions

This meta-analysis shows that MI combined with CRT exerts a small-to-moderate but uncertain effect on upper-limb motor recovery after stroke. However, this apparent benefit diminishes substantially when accounting for methodological flaws and publication bias. The observed improvements fall below the MCID threshold, limiting their clinical relevance. Heterogeneous protocols, non-standardized MI dosing, and the lack of systematic assessment of imagery ability further weaken the evidence base. Current data therefore does not support MI as an effective adjunct to CRT, although it may hold promise within integrated and technology-enhanced rehabilitation programs, such as those incorporating virtual reality, BCI-FES, or NIBS. Larger and methodologically rigorous RCTs with standardized MI protocols are needed to determine their true therapeutic value in post-stroke rehabilitation.

## Figures and Tables

**Table 1:** Baseline Characteristics.

Study	Study Design	Phase Stroke	Etiology	Lesion laterality	Severity FM-UE	Groups	Age	Intervention	Intervention volume			Outcomes	Results	
									Weeks	Frequency	Session Duration (Minutes)			
[43]	Pilot RCT	Subacute	Ischaemic and haemorrhagic	Both	Severe	IG (12)	61.8	MP combined with CRT (proprioceptive exercises, gait training, hand and wrist mobilization, stretching, weight bearing, strengthening, and functional task practice).	4	5	MI: 20 CRT: 30	6FM-UE MFT FIM	No significant differences were found between MP plus CRT alone in subacute post-stroke patients.	
					Severe	CG (12)	59.6	CRT	4	5	30			
[50]	RCT	Chronic	Ischaemic and haemorrhagic	Both	Near Normal	IG (15)	47.5	MI with the imagery guided by an audio tape and CRT.	3	4	MI: 15 CRT: 15	MAL-AOU MAL-QOM FM-UE	Participation in a MI protocol can improve the upper extremity function in chronic stroke patients.	
					Near Normal	CG (15)	50.1	CRT	3	4	15			
[44]	Crossover	Subacute	Ischaemic and haemorrhagic	Both	Mild	IG (5)	57.9	MP protocol including two tasks (drinking from a cup and opening a door) in addition to CRT.	3	MI: 3 CRT: 5	MI: 20 CRT: 30	FM-UE MAL-AOU MAL-QOM 3D motion analysis	Adjuvant MP showed no significant effects on upper limb function after stroke.	
					Mild	CG (5)	57.9	CRT.	3	5	30			
[51]	RCT	Subacute and chronic	Ischaemic	Both	Moderate	IG (8)	64.4	CRT including upper and lower limb exercises, transfers, balance/walking training, and activities of daily living performed bimanually, combined with guided MI sessions after each therapy.	6	3	MI: 10 CRT: 60	FM-UE ARAT	MI was a feasible and cost-effective complement to therapy, improving outcomes compared to therapy alone.	
					Moderate	CG (5)	65.0	CR including the same program of upper and lower limb exercises, transfers, balance/walking training, and activities of daily living	6	3	60			
[46]	RCT	Chronic	Ischaemic and haemorrhagic	Both	Moderate	IG (16)	58.7	CRT focused on activities of daily living, combined with daily MP sessions directly after therapy.	6	2	MI: 30 CRT: 30	FM-UE ARAT	MP programs significantly improved arm motor function in chronic stroke patients.	
					Moderate	CG (16)	60.4	CRT with equal therapist interaction.	6	2	CRT: 30			
[47]	RCT	Chronic	Ischaemic and haemorrhagic	Right hemisferic	Moderate	IG (14)	60	MI focused on daily tasks (e.g., page turning, bean transfer, cup stacking) and CRT.	2	5	MI:10 CRT: 30	ARAT FM-UE MBI	MI improved upper extremity function and daily activity performance in stroke patients.	
					Low—Moderate	CG (15)	58	CRT.	2	5	30			
[48]	RCT	Chronic	Ischaemic and haemorrhagic	Both	Severe	IG (9)	56.7	Standard CRT —including physical and occupational therapy, electrical stimulation, acupuncture, and massage—supplemented with MI training.	4	5	MI:30 CRT: 180	FM-UE	MI induced cortical reorganization in chronic stroke patients, supporting motor function improvement.	
					Severe	CG (9)	56.1	Standard CRT	4	5	180			
[45]	RCT	Subacute	Ischaemic and haemorrhagic	Both	Severe	IG (13)	58.6	CRT (physical therapy, occupational therapy, electrical stimulation, and Chinese acupuncture) plus specific MI training	4	5	MI: 30 CRT: 120	MBI FM-UE	MI training combined with CRT significantly improved upper limb function and daily activities compared to rehabilitation alone.	
					Severe	CG (13)	60.2	CRT (physical therapy, occupational therapy, electrical stimulation, and Chinese acupuncture)	4	5	120			
[49]	RCT	Chronic	Ischaemic and haemorrhagic	Both	Severe	IG (17)	53.4	CRT supplemented with supervised MI training of the affected upper limb—including relaxation, basic movements, and goal-directed daily activities	4	5	MI:30 CRT: 180	FM-UE MBI fMRI	MI training significantly improved FM-UE compared to CG, accompanied by increased fractional amplitude of low-frequency fluctuations (slow-5) and altered functional connectivity in the ipsilesional inferior parietal lobule, both correlated with motor recovery.	
					Severe	CG (17)	60.5	CRT	4	5	CRT: 180			
[52]	RCT	Subacute and chronic	Ischaemic and haemorrhagic	Both	Severe	IG (22)	54.4	MI of the upper limb with first-person practice of movements and daily activities, added to CRT (physical therapy, occupational therapy, neuromuscular electrical stimulation, and acupuncture).	4	5	MI: 30 CRT 180	MBI FM-UE	MI training improved motor recovery beyond CRT, with greater benefits in patients with impaired motor planning but preserved motor imagery ability.	
					Severe	CG (17)	59.7	CRT included physical therapy, occupational therapy, neuromuscular electrical stimulation, and Chinese acupuncture.	4	5	CRT: 180			

Abbreviations: ARAT, Action Research Arm Test; CG, Control Group; CRT, Conventional Rehabilitation Therapy; FIM, Functional Independence Measure; FM-UE, Fugl-Meyer Upper Extremity; fMRI, Functional Magnetic Resonance Imaging; IG, Intervention Group; MAL-AOU, Motor Activity Log—Amount of Use; MAL-QOM, Motor Activity Log—Quality of Movement; MBI, Modified Barthel Index; MFT, Manual Function Test; MI, Motor Imagery; MP, Mental Practice; RCT, Randomized Clinical Trial.

**Table 2:** Methodological score of RCTs using the PEDro scale.

Study	1	2	3	4	5	6	7	8	9	10	11	Total
[51]	Y	Y	N	N	N	N	Y	Y	Y	Y	Y	6
[46]	Y	Y	N	Y	N	N	Y	Y	Y	Y	Y	7
[47]	Y	Y	N	Y	N	N	N	Y	N	Y	Y	5
[45]	Y	Y	N	Y	N	N	Y	N	N	Y	Y	5
[52]	Y	Y	N	Y	N	N	Y	Y	Y	Y	Y	7
[44]	Y	Y	N	Y	N	N	N	Y	Y	Y	Y	6
[48]	Y	Y	N	Y	N	N	Y	Y	N	N	Y	5
[49]	Y	Y	N	Y	N	N	Y	Y	N	N	Y	5
[50]	Y	Y	N	Y	N	N	N	Y	Y	Y	Y	6
[43]	Y	Y	N	Y	N	N	Y	Y	N	Y	Y	6

**Table 3:** Summary of the GRADE assessment.

Studies	Risk of Bias	Inconsistency	Indirectness	Imprecision	Publication Bias	SMD (95% CI)	Quality
10 RCTs (*n* = 255)	Very serious	Serious (I^2^ = 88.2%)	No serious	Serious	Very serious	ES = 0.45 (0.16, 0.74) Adjusted ES = −0.06 (−0.21, −0.08)	Very Low

## Data Availability

All data extracted from the included trials and used in this meta-analysis are openly available in the Supplementary Excel File provided with this manuscript. Statistical analyses were conducted in R software using the metafor and orchaRd packages. The complete R code applied for the analyses is also included in the Supplementary Materials to ensure full reproducibility.

## Supplementary Materials

The following supporting information can be downloaded at: https://www.mdpi.com/article/10.3390/jcm14217891/s1, Supplementary Material S1: PRISMA 2020 checklist; Supplementary Material S2: Search strategy and Boolean operators used in each database; Supplementary Material S3 and S4: Excel file containing all extracted data from the included studies. Statistical analyses were conducted in R software using the metafor and orchaRd packages, and the complete R code applied for the analyses is included to ensure full reproducibility.

## Abbreviations

The following abbreviations are used in this manuscript:

ARAT	Action Research Arm Test
BBT	Box and Block Test
BCI-FES	Brain–Computer Interface with Functional Electrical Stimulation
CG	Control Group
CRT	Conventional Rehabilitation Therapy
ES	Effect Size
FM	Fugl-Meyer Assessment
FM-UE	Fugl-Meyer Assessment—Upper Extremity
fMRI	Functional Magnetic Resonance Imaging
fNIRS	Functional Near-Infrared Spectroscopy
GRADE	Grading of Recommendations Assessment, Development, and Evaluation
ICC	Intraclass Correlation Coefficient
IG	Intervention Group
I^2^	I-squared heterogeneity statistic
MCID	Minimal Clinically Important Difference
MI	Motor Imagery
MP	Mental Practice
NIBS	Non-Invasive Brain Stimulation
PEDro	Physiotherapy Evidence Database scale
PICOS	Population, Intervention, Comparison, Outcomes, Study design
PRISMA	Preferred Reporting Items for Systematic Reviews and Meta-Analyses
PROSPERO	International Prospective Register of Systematic Reviews
RCT	Randomized Controlled Trial
REML	Restricted Maximum Likelihood
RoB	Risk of Bias
SMC	Standardized Mean Change
SMD	Standardized Mean Difference
